# Differential Effects of Friendship and School Norms on Adolescents’ Defending in Cyberbullying Situations: A Randomized School-Based Experiment

**DOI:** 10.1007/s10964-025-02202-y

**Published:** 2025-06-11

**Authors:** Stefanie Richters, Maor Shani, Lea Geyer, Maarten H. W. van Zalk

**Affiliations:** Developmental Psychology, Institute for Psychology, Osnabrück University, Lise-Meitner-Str. 3, 49076 Osnabrück, Germany

**Keywords:** Cyberbullying, Defending, Social norms, Friendships, Adolescence

## Abstract

During adolescence, close friendships become increasingly important, yet whether norms within friendship groups may influence responses to cyberbullying more than norms from more distal reference groups such as schools remains untested. This preregistered experiment examined the effects of friendship and school pro-defending norms on adolescents’ defending intentions and behaviors in response to hypothetical cyberbullying scenarios. Participants were 321 students from grades 5 to 10 in a German secondary school (55.45% female; *M*_age_ = 12.66, *SD*_age_ = 1.73), randomly assigned to a friendship norm (*N* = 105), school norm (*N* = 110), or control (*N* = 106) condition. Norm information was derived from previous data collection. Victim- and bully-oriented defending intentions and behaviors were significantly higher in the friendship norm condition compared to the control condition, while the school norm condition showed no significant effects. Neither norm condition influenced cyberbullying reporting and participation in an anti-bullying campaign. These findings demonstrate that friendship norms are effective in promoting defending in cyberbullying situations, suggesting that norm-based interventions may benefit from targeting such proximal reference groups that are aligned with adolescents’ developmental needs.

## Introduction

Cyberbullying in schools is a widespread problem with serious negative short- and long-term consequences for the actors involved (Flannery et al., 2023). In Germany, cyberbullying is a highly prevalent issue: recent data show that 18.5% of adolescents have experienced cyberbullying, a figure that has risen over the past years (Bündnis gegen Cybermobbing, 2024). Most adolescents (93%) own a smartphone, and 96% use WhatsApp daily (Feierabend et al., 2024), making online communication a central feature of adolescent life. (Mis-)perceptions of social norms play an important role in adolescents’ behavior in bullying (Shin & Gyeong, 2024) and cyberbullying situations (Piccoli et al., 2020). Targeting perceptions of norms can be an effective strategy to create behavioral change (e.g., Miller & Prentice, 2016). To date, most norm interventions in the school context have targeted school-wide or classroom-specific norms. However, during adolescence, friendships have a strong influence on prosocial and problematic development (Veenstra & Laninga-Wijnen, 2023). The purpose of the present study is to examine the influence of friendship versus school pro-defending norms on defending in cyberbullying scenarios. This will be examined through a school-based experiment with adolescents in Germany.

### Social Influence Through Norms

The term social norm is commonly used to describe two different phenomena: first, it refers to the typical or average behavior within a group (descriptive norm). Second, it refers to beliefs about what behavior is expected or approved of in a group (injunctive or prescriptive norm; Cialdini et al., 1990). In addition, it is important to distinguish between actual (descriptive or prescriptive) and perceived norms: because people typically don’t have a representation of actual opinions and behaviors within a group, their own opinions and behaviors may be guided by their perceptions of the norm (Tankard & Paluck, 2016).

Work in line with the group norms theory (Sherif & Sherif, 1953) suggests that people are motivated to adopt attitudes and behaviors expressed by others who represent important group identities, because they are motivated to feel socially connected to their group (e.g., Paluck, 2011). The social identity theory (Tajfel & Turner, 1979) also addresses the influence of group membership on behavior, predicting that normative influence will be stronger when group membership is self-defining and contextually salient than when group membership is perceived as less relevant to one’s self-concept or less prominent in the given context (Schultz, 2022). Taken together, the source of normative information (i.e., the reference group) is critical to the influence of such information on one’s behavior: the norms of reference groups with which an individual strongly identifies and values are particularly likely to guide behavior (Prentice, 2012).

### The Experimental Context: Cyberbullying Prevention

Cyberbullying is commonly defined as bullying which takes place via mobile phones or the internet, characterized by intentional and repeated aggressive actions targeting victims who are not able to defend themselves (Smith et al., 2008). Similar to traditional bullying, cyberbullying can be understood as a group process in which not only the behavior of the bullies, but also that of the bystanders is important: bystanders can remain silent, reinforce the bullying or take side with the victim (Sarmiento et al., 2019). Defending in cyberbullying situations can be defined as a prosocial response by bystanders and can take various forms which include comforting the victim, counterattacking the bully and reporting the cyberbullying incident (Chen et al., 2024).

(Mis-)perceptions of social norms play an important role in adolescents’ reactions to traditional bullying (Shin & Gyeong, 2024) as well as cyberbullying situations (Piccoli et al., 2020). A plausible explanatory mechanism why only few adolescents engage in defending is *pluralistic ignorance*: adolescents misinterpret their peers’ inaction in bullying situations as a prescriptive norm supportive of bullying, which then contributes to their own inaction and to maintaining the status quo of low defending over time (Shin & Gyeong, 2024). To promote defending in bullying situations, pluralistic ignorance can be reduced by providing adolescents with accurate information about prescriptive norms within their group. Previous interventions using this approach to reduce traditional bullying have presented (anti-)bullying school norms (Perkins et al., 2011), grade-level norms (Dillon & Lochman, 2022), and classroom norms (Tolmatcheff et al., 2022). These interventions have shown promising results, leading to a reduction of bullying, victimization and perceptions of pro-bullying attitudes (Perkins et al., 2011); a significant decrease in bullying (Tolmatcheff et al., 2022); and a reduction in norm misperception (Dillon & Lochman, 2022).

However, the application of norm-based interventions to cyberbullying remains limited and less conclusive. A study conducted in the Netherlands with adolescents aged 11-16 found that presenting information about a generalized social norm against cyberaggression (“Most people disapprove of cyber aggressive behaviors”) failed to reduce conformity to such behaviors (Bleize et al., 2022). In a sample of German adolescents (grade 7 to 9), providing information about classroom anti-bullying norms had no effect on individual attitudes (Pfetsch et al., 2018). One possible explanation is that the classroom may constitute a less salient reference group in cyber contexts, where social interactions often transcend classroom boundaries (Pfetsch et al., 2018).

A meta-analysis of social norm interventions highlights an important limitation in the existing literature: most norm-based interventions rely on distal reference groups, while proximal reference groups remain underexamined, limiting the ability to draw meaningful comparisons (Rhodes et al., 2020). This is surprising given strong theoretical accounts suggesting that norms are most influential when endorsed by groups with which individuals strongly identify (Prentice, 2012). From this perspective, it remains unclear whether the school norm, as targeted in the original anti-bullying norm intervention study (Perkins et al., 2011) and other large-scale norm intervention studies (e.g., Paluck et al., 2016), is the best reference group to target. Although these school norm interventions have demonstrated positive effects on bullying-related outcomes, their impact may be constrained by the relatively distal nature of the school as a reference group. Targeting norms of more proximal reference groups, such as close friends, may enhance the effectiveness of norm-based interventions – particularly in adolescence, when friendships become increasingly important (Veenstra & Laninga-Wijnen, 2023).

Adolescence is a period characterized by shifting social priorities and declining responsiveness to adult-led interventions (Yeager et al., 2015). A meta-analysis showed a substantial drop in the effectiveness of anti-bullying programs with participants from the age of 14, showing that programs that are effective in childhood are less so during adolescence (Yeager et al., 2015). This decline is likely rooted in adolescents‘ growing need for autonomy and independence from adult authorities (Yeager et al., 2018). Concurrently, adolescents become increasingly sensitive to peer influence (Steinberg & Monahan, 2007), and the effectiveness of social norm interventions is largest during this developmental period (Rhodes et al., 2020). Peer-led interventions can be particularly effective in adolescence, because they align with adolescents’ developmental needs, enhance information retention, and facilitate lasting normative changes (Veenstra, 2025).

Consistent with the heightened salience of friendships during adolescence, several observational studies have shown the significance of friendship norms for cyberbullying dynamics. Adolescents who believe their friends are involved in cyberbullying, are more inclined to engage in such behaviors themselves (Hinduja & Patchin, 2013). Adolescents who perceive their friends to approve of cyberbullying are more likely to join the cyberbullying through mechanisms of social pressure, whereas perceived class-level approval shows no significant effect (Bastiaensens et al., 2016). In addition, strong endorsement of pro-cyberbullying descriptive norms within friendship groups is associated with higher cyberbullying rates (Piccoli et al., 2020). Recent work has extended the social identity perspective to cyber contexts, showing that adolescents’ identification with WhatsApp group members indirectly predicts conformity to cyber aggression via perceived social pressure to conform to group norms (Bleize et al., 2021). Despite these findings, no study to date has assessed the effectiveness of providing accurate information about friendship norms in a social norm intervention to reduce cyberbullying, nor compared the influence of norms from different reference groups on cyberbullying.

The design of social norm interventions requires careful attention to several theoretically grounded decisions that can significantly influence their effectiveness. One key aspect is the distinction between prescriptive and descriptive norms. Meta-analytic evidence suggests that prescriptive norms are more effective in shaping behavior (Rhodes et al., 2020). Accordingly, the present study employed prescriptive norm messages. Moreover, whereas prior interventions have focused on anti-bullying norms, the current study shifts focus on pro-defending norms. Encouraging defending among bystanders and discouraging bullying behavior can be understood as two distinct intervention pathways. Norm interventions are applied at the group level, where the focus of the intervention is typically on encouraging bystanders to defend (Salmivalli, 2014). Thus, providing information about pro-defending rather than (anti-)bullying norms may be a more direct and effective way to promote defending. Analyses of pre-intervention data showed that pluralistic ignorance with respect to defending norms occurs at both the school and friendship group level: students perceive their peers (within their school and their friendship group) to be less supportive of defending than they actually are (see the Online Resource 1). This result suggests that providing information about prescriptive pro-defending norms can be an effective strategy to promote defending.

In addition, individual demographic characteristics may shape adolescents’ responses to bullying and cyberbullying, as well as their susceptibility to normative influence, thereby potentially moderating the effectiveness of norm-based interventions like the one examined in this study. Gender differences in defending are well established, with girls consistently more likely than boys to defend victims (Ma et al., 2019). As adolescents grow older, defending tends to decline (Ma et al., 2019), possibly due to increasing reputational concerns (Laninga-Wijnen & Veenstra, 2021). Migration background may further influence defending and the perceived relevance of different normative messages (e.g., from the school vs. friends) through its effects on group identification. For example, differential identification with potentially diverse school communities versus more homogenous friendship groups could affect how adolescents from various backgrounds internalize and act upon pro-defending norms presented from these sources, in line with findings that adolescents are more likely to defend same-ethnic peers than cross-ethnic peers (Hooijsma et al., 2021).

## Current Study

Social norms shape adolescents’ behavior in cyberbullying situations, but it remains untested whether norms from schools and friendship groups differentially affect their defending responses. The first aim of this study was to test whether presenting adolescents with prescriptive pro-defending norms from either school or friendship groups would lead to increased defending intentions and actions compared to a control group. Given the promising results of previous norm intervention studies, participants who were presented with prescriptive pro-defending norms from school or friendship groups were expected to show higher defending intentions and actions compared to the control condition (Hypothesis 1). Specifically, it was hypothesized that defending intentions (comforting the victim; confronting the bully; reporting the bullying) and defending (by sending a message to the victim and the bully) in hypothetical scenarios, as well as the participation in an anti-bullying poster campaign would be higher after presenting prescriptive norms showing high agreement with defending (in school and in friendship groups) compared to the control group. The second aim of this study was to test whether defending intentions and actions are higher when friendship norms, compared to school norms, are presented. Due to the increased importance of friendships during adolescence, friendship norms were expected to be more influential than school norms (Hypothesis 2). Specifically, it was hypothesized that defending intentions (comforting the victim; confronting the bully; reporting the bullying) and defending (by sending a message to the victim and the bully) in hypothetical scenarios, as well as the participation in an anti-bullying poster campaign would be higher after presenting prescriptive norms showing high agreement with defending in the friendship group compared to presenting prescriptive norms showing high agreement with defending in the school. In addition to the preregistered analyses, exploratory models included gender, age, and migration background as covariates, along with their interactions with condition, to examine whether the effects of the norm presentation differed across demographic subgroups.

## Methods

This study’s design, hypotheses and analyses were preregistered at https://osf.io/8wrcs. The study materials, data, analysis code and results output are available at https://osf.io/a2mf7.

### Study Design

The study was part of a larger research project which involved four waves of data collection taking place between November 2021 and June 2022 (wave 1: November 2021; wave 2: February 2022; wave 3: May 2022; wave 4: June 2022). A school-based experiment was conducted as part of the fourth wave of the data collection. The norm information was based on real attitudinal information collected in the second and third waves of the data collection.

The experiment employed a between-subjects design in which the presentation of prescriptive defending norms was experimentally manipulated. Participants were assigned to one of three conditions:

1. presentation of school-wide prescriptive defending norms
2. presentation of friendship prescriptive defending norms
3. no norm presentation (control group)

For ethical reasons, only true information about prescriptive defending norms was presented. As a consequence, participants were included in the analyses only if they were eligible for the friendship norm condition, which required meeting the following criteria: (1) They participated in at least one wave of the previous data collection and thus could nominate their friends prior to the experiment. (2) They reported at least three friends in either wave of the previous data collection and thus had a sufficiently large friendship group. (3) their friendship group met the criteria for reporting positive prescriptive defending norms (see details below). See the Online Resource 2 for additional analyses and more details about the causes and consequences of these inclusion criteria.

Participants were randomly and with equal probability assigned to one of the three conditions using the survey software SoSciSurvey. Participants who were assigned to, but not eligible for, the friendship norm condition were re-assigned randomly and with equal probability to one of the two other conditions. See the preregistration for details on the implementation of the randomization.

Following the experimental manipulation, participants were presented with two cyberbullying scenarios to assess their likelihood of intervention (i.e., behavioral intention), their responses to the hypothetical scenarios (i.e., writing a message to the actors involved), and their participation in an anti-bullying poster campaign in their school, which served as a measure of actual behavior.

### Sample

Data were collected in a secondary school (Oberschule) in Lower Saxony, Germany. This school type combines elements of lower- and middle-tier education tracks and is typically attended by students with varying academic achievement levels. All students (grades 5 to 10) were invited to participate. The initial sample consisted of 496 participants (50.60% female; 22.78% with migration background, defined as participant and/or at least one parent born outside Germany) aged between 10 to 17 years (*M*_age_ = 12.67, *SD*_age_ = 1.73). Two participants did not respond to any of the outcome variables and were therefore excluded from the analyses. Further, participants who were not eligible to the friendship norm condition were excluded from the analyses. The final sample consisted of 321 participants (55.45% female; 20,25% with migration background) between the ages of 10 and 17 years (*M*_age_ = 12.66, *SD*_age_ = 1.73). Table 1 presents the distribution of students across ages. 106 participants were assigned to the control condition, 110 to the school norm condition and 105 to the friendship norm condition. No missing data were observed on any of the study variables within the final analytic sample.

**Table 1:** Distribution of participants by age

age (in years)	10	11	12	13	14	15	16	17
frequency (*N*)	35	65	50	71	44	39	15	2
proportion (%)	10.90	20.25	15.58	22.12	13.71	12.15	4.67	0.62

Total *N* = 321.

To assess potential selection effects, comparisons were conducted between the final analytic sample and participants who were excluded due to ineligibility to the friendship norm condition. An independent samples t-test indicated that there is no significant difference in age between the groups (*t*(352.63) = 0.29, *p* = 0.769). The proportion of girls is higher in the final sample (55.80%) compared to the excluded group (42.11%). A chi-square test confirmed that this difference is statistically significant (*χ*^*2*^(1, *N* = 490) = 7.81, *p* = 0.005). Further, the proportion of students with migration background is smaller in the final sample (20.25%) compared to the excluded group (27.75%), but this difference is not significant (*χ*^*2*^(1, *N* = 494) = 3.17, *p* = 0.075).

Results of an a priori power analysis using G*Power version 3.1 (Faul et al., 2007) indicated that for repeated measures, between factors MANOVAs with an effect size *f* = .25, α = .05 and power (1-β error probability) = .80 the required total sample size is *N* = 102. Thus, the expected sample size was adequate to test the hypotheses.

### Procedure

The study was approved by the ethical committee of the corresponding writer’s institute. Prior to the start of the project, both legal guardians (e.g. parents) and students received detailed written information about the study. The materials provided to students were age-appropriate and tailored to ensure comprehension. Legal guardians and students themselves were both required to provide active informed consent for participation. Data collection took place during school hours. Trained research assistants verbally explained the study procedures to students using accessible age-appropriate language. Students were encouraged to ask questions and were informed that participation was entirely voluntary. After these explanations, students were asked to provide active assent to participate before beginning the questionnaire. For practical reasons, participants completed the questionnaire on their own smartphone. Participants who did not have a smartphone or preferred not to use their own device were provided with a smartphone for the duration of the data collection session. This approach allowed for efficient distribution and completion of the survey, minimized the need for paper materials, and facilitated direct data entry into the study database. Participants received standardized instructions at the beginning of the questionnaire and could ask questions at any time during the session. Participants who were absent from school during the data collection session could participate in an online session later. Four participants took part in the online session.

### Experimental manipulation

**Fig. 1:**
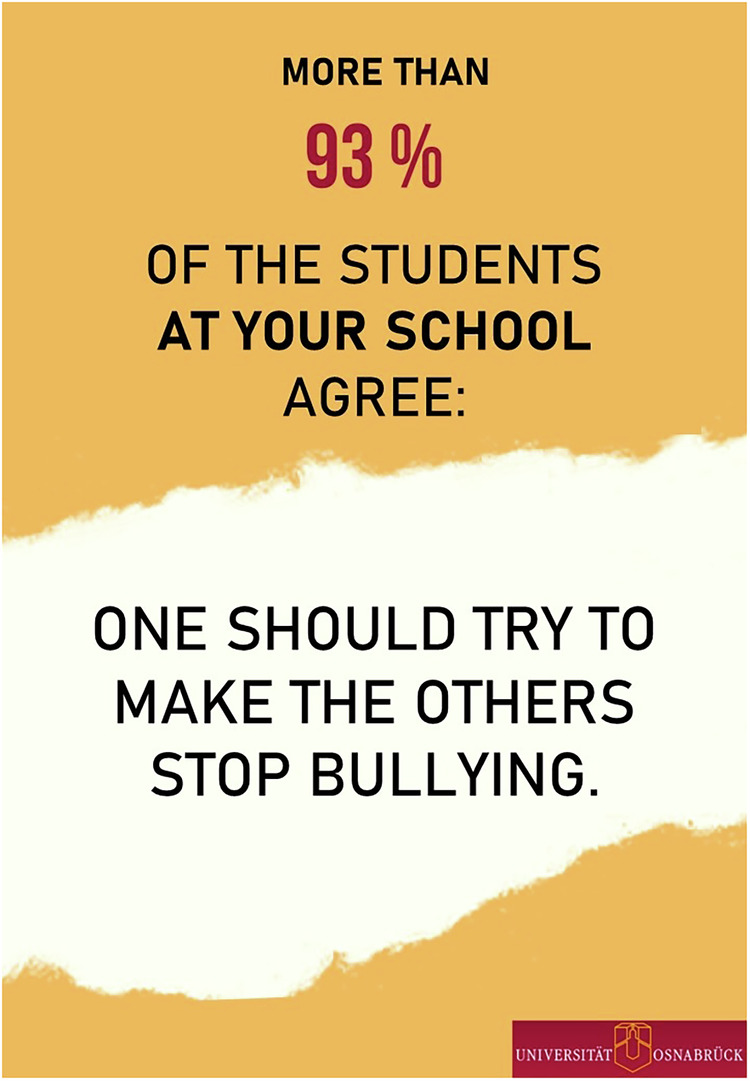
Example of the norm information provided in the school norm condition. In the friendship norm condition, “of the students at your school” was replaced by “of your friends”

In the school norm and friendship norm conditions, participants were presented with information about victim- and bully-oriented prescriptive defending norms within the respective group (see Fig. 1 for an example). These were based on the average scores of participants’ entire school community or their friendship groups in the second and third waves of data collection on two items measuring attitudes toward defending: “One should comfort the victim of bullying afterwards.” (victim-oriented defending) and “One should try to make the others stop the bullying.” (bully-oriented defending). Both items were assessed on a 5-point scale ranging from 1 = “fully disagree” to 5 = “fully agree”.

For the school norm information, the percentage of participants in the school who agreed with the two items (i.e., they scored “4 = rather agree” or “5 = fully agree”) were calculated per wave and the percentage was averaged across waves two and three. This resulted in 95.57% agreement for victim-oriented defending and 93.15% agreement for bully-oriented defending.

For the friendship norm condition, the percentage of the nominated friends who agreed with the two items was calculated for each student. A student’s friendship group was defined as all participants who were nominated as friends by that person. Friendship nominations did not need to be reciprocal, as the significance of these peers to the participants was the focus, regardless of reciprocity. When data from both waves were available, the mean percentage was calculated. Importantly, to isolate the effect of the reference group (i.e., school norm versus friendship norm), the agreement percentage was kept identical across the conditions at the level of the school-wide agreement. This design choice ensured that any observed effects could be attributed solely to the reference group, rather than differences in the level of agreement. For ethical reasons, only true norm information was provided. The norm information was phrased as: “More than 95% or the students at your school [or: of your friends] agree…”. In order to truthfully present this message in the friendship norm condition, the participant’s friendship group had to meet or exceed the school-wide agreement threshold. Due to this constraint, participants whose friendship groups had a lower agreement rate than the average school agreement (i.e., 95% for victim-oriented defending and 93% for bully-oriented defending) were not eligible for the friendship norm condition and were excluded from the analysis. On average, the excluded participants’ friendship groups had agreement rates of 82.10% for victim-oriented defending and 81.30% for bully-oriented defending. A detailed description of how the norm information was calculated (including Rscripts) is available in the preregistration.

### Measures

#### Defending intentions

Participants were presented with two hypothetical cyberbullying scenarios, illustrated as screenshots of Instagram stories. In one scenario, participants were instructed to imagine encountering the story in their own Instagram feed. In the other, the screenshot was described as having been sent by a friend. In both scenarios, participants were asked to assume that the individuals involved were classmates. The instructions included brief contextual details indicating that the victims were socially vulnerable and had previously been targeted by peers. Following the presentation of each scenario, participants were asked “How do you react?” and were presented with a series of possible responses to the scenarios. For each response option, they were asked to rate the likelihood of them engaging in it on a scale ranging from 1 (very unlikely) to 6 (very likely). For each scenario, the likelihood of engaging in three distinct types of defending was assessed: (1) *comforting the victim* by sending him/her a direct message; (2) *confronting the bully* by sending him/her a direct message; and (3) *reporting the cyberbullying* using Instagram’s reporting feature. The two additional response options were included to reflect a broader range of possible responses (not reacting; liking or reposting the story) but were not the focus of this study. All response options were rated independently, allowing participants to indicate a high likelihood of engaging in multiple responses. For each type of defending, mean scores across the respective items in the two scenarios were computed. Pearson correlations between the two items were moderate to strong with *r* = 0.56 (*p* < 0.001) for the sub-scale *comforting the victim*; *r* = 0.44 (*p* < 0.001) for the sub-scale *confronting the bully*; *r* = 0.63 (*p* < 0.001) for the sub-scale *reporting the cyberbullying*.

#### Messages to the victim and bully

If participants rated their likelihood of comforting the victim and/or confronting the bully as 4 or higher on the behavioral intentions scale, they were prompted with a follow-up question in which they were given the opportunity to compose an actual *message to the victim* and/or *message to the bully*. Their written responses were then coded by two independent coders to determine whether they represented defending. The criteria for this classification were derived from the ‘defender scale’ of the Peer Relations Questionnaire (Salmivalli et al., 1996; see preregistration for details). Responses were coded as 1 if they represented defending, non-defending responses were coded as 0. Inter-coder reliability was very high for both the *message to the victim* (κ = 0.98 in scenario 1; κ = 0.97 in scenario 2) and for the *message to the bully* (κ = 0.97 in scenario 1; κ = 0.97 in scenario 2). Spearman rank correlations between the coded responses across the two scenarios were moderate for the *message to the victim* (*ρ* = 0.42) and for the *message to the bully* (*ρ* = 0.43). Sum scores for the coded responses across the two scenarios for the two sub-scales were computed, resulting in two ordinal-scaled scores with 0 = no defending; 1 = moderate level of defending; 2 = high level of defending.

#### Participation in an anti-bullying poster campaign

Participants participation in an anti-bullying poster campaign was assessed as a measure of actual behavior. As part of the questionnaire, participants could submit content that was later used to create posters. These posters were designed by one of the co-authors and delivered to the school after all data collections were finalized. They were publicly displayed in the school, making them visible to the entire student body, although only study participants contributed content. All participants, regardless of age, received standardized instructions, informing them that their responses will be displayed publicly at school and emphasizing the opportunity to contribute to school-wide anti-bullying messaging. Participants could contribute to the campaign in three distinct ways: (1) signing a poster with their name as a supporter of the campaign; (2) writing a personal statement on bullying; (3) signing their personal statement with their name. For each of the three options, a variable was coded as 1 for participation and 0 for non-participation. The variable relating to the personal statement was coded by two independent coders. Inter-rater reliability was very high (κ = 0.916). The internal consistency between the three items was high (ordinal α = 0.93). A sum score of the three coded variables was created, resulting in ordinal scales scores with 0 = no participation; 1 = low level of participation; 2 = moderate level of participation; and 3 = high level of participation.

#### Comprehension check

A factual manipulation check (Kane & Barabas, 2019) with two single-choice questions was administered at the end of the questionnaire to all students assigned to the school norm and friendship norm conditions (*N* = 215). Overall, 51% of these participants did not answer at least one of the two questions as expected (question 1: 32%; question 2: 33%). However, the first question contained a mistake in the wording of the answer options. Participants were asked “Whose average answers did we present to you?” with response categories “results from your friends” and “results of the students in your school”. As the friends referred to in this study are always students in their school, these two options were not mutually exclusive and did not allow for a clear distinction between the experimental conditions. As a result, only the second comprehension check question was retained. In this second question, participants were asked “How large was the agreement with the statements that were presented to you?” with the response options “less than 10% (few people agreed)”, “about 50% (about half agreed)” and “more than 90% (almost everyone agreed)”. 33% of participants selected the incorrect response for their condition.

#### Demographic measures

Gender was coded as 1 = “girl” and 2 = “boy”. Responses from students who selected “diverse” (< 0.01%) were coded as missing due to the small sample size. Age was assessed in years. Migration background was determined based on participants’ self-reported place of birth as well as that of their parents. Participants were coded as 1 = having a migration background if they themselves and/or at least one parent were born outside of Germany, else they were coded 0.

### Analyses

All analyses were carried out using R (version 4.2.2). To test whether the experimental condition affected defending intentions, a one-way MANOVA with experimental condition as fixed factor and three dependent variables (one per type of defending intention) was conducted. Following a significant F-test obtained in the MANOVA, follow-up univariate ANOVAs were conducted. In case of significant univariate ANOVAs, post-hoc comparisons between the pairs of groups were performed with Tukey correction to the p-value.

Ordinal logistic regressions were conducted to test whether the experimental condition affected bully- and victim-oriented defending in hypothetical scenarios, and participation in an anti-bullying poster campaign using the polr function of the MASS package in R. To test Hypothesis 1, it was examined whether the scores in the two experimental conditions differed significantly from the scores in the control condition (comparison group). To test Hypothesis 2, the conditions were re-leveled with the school norm condition as the comparison group to examine whether scores in the friendship norm condition differed significantly from scores in the school norm condition (this second step was missing in the preregistration of the analyses and was added to test Hypothesis 2).

In addition to the primary analyses, a series of robustness checks were conducted to assess the stability and generalizability of the findings. First, the main analyses were repeated using a reduced sample that excluded participants who failed the valid comprehension check item. This approach is in line with recommendations (Hauser et al., 2018). Second, models were estimated that included participants’ gender, age and migration background as covariates, along with interaction terms between each covariate and the experimental condition. This allowed for the examination of whether the intervention effects were consistent across subgroups and whether any effects were moderated by demographic characteristics.

## Results

Table 2 presents a summary of the key findings.

**Table 2:** Summary of the key findings

Outcome	friendship norm vs control	school norm vs control	friendship norm vs school norm
Intention to comfort the victim	↑ (*p* < 0.001, *d* = 0.51)	ns (*p* = 0.323)	ns (*p* = 0.068)
Intention to confront the bully	↑ (*p* = 0.035, *d* = 0.36)	ns (*p* = 0.933)	ns (*p* = 0.078)
Intention to report the bullying incident	ns	ns	ns
Messages to the victim	↑ (*p* = 0.025, *OR* = 1.81)	ns (*p* = 0.461)	ns (*p* = 0.132)
Messages to the bully	↑ (*p* = 0.037, *OR* = 1.71)	ns (*p* = 0.420)	ns (*p* = 0.192)
Participation in the anti-bullying poster campaign	ns (*p* = 0.835)	ns (*p* = 0.216)	ns (*p* = 0.167)

↑ significantly higher compared to the reference group; ns non-significant; *d* Cohen’s d; *OR* odds ratio

### Effects of the Experimental Condition on Defending Intentions

The one-way MANOVA revealed an overall significant effect of experimental condition on the combined dependent variables of defending intentions (*F*(6, 634) = 2.60, *p* = 0.017, *η*_*p*_^*2*^ = 0.02). Follow-up univariate ANOVAs showed that the experimental condition had a significant effect on intentions to comfort the victim (*F*(2, 318) = 6.69, *p* = 0.001, *η*_*p*_^*2*^ = 0.04) and on intentions to confront the bully (*F*(2, 318) = 3.67, *p* = 0.027, *η*_*p*_^*2*^ = 0.02), but not on intentions to report the cyberbullying via the Instagram reporting feature (*F*(2, 318) = 1.93, *p* = 0.147).

Intentions to comfort the victim were highest in the friendship norm condition (*M* = 4.88, *SD* = 1.20), followed by the school norm condition (*M* = 4.47, *SD* = 1.43) and control group (*M* = 4.20, *SD* = 1.43). Post-hoc comparisons revealed a significant mean difference between the friendship norm condition and the control condition (*p* < 0.001, *d* = 0.51). Mean differences between the school norm condition and control condition (*p* = 0.323), as well as the friendship norm condition and the school norm condition (*p* = 0.068) were non-significant.

**Fig. 2:**
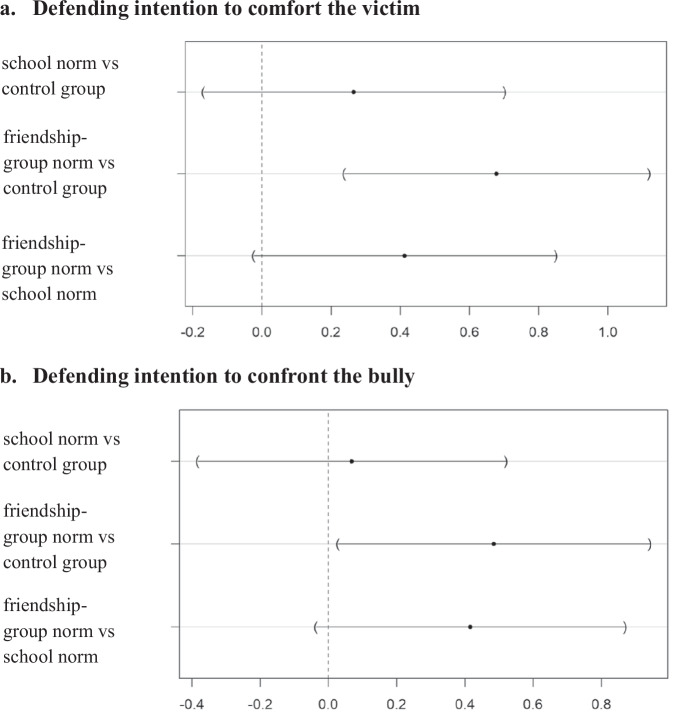
Mean differences between pairs of experimental conditions with 95% confidence intervals (**a**) for the intention to comfort the victim and (**b**) the intention to confront the bully

Intentions to confront the bully were overall lower compared to intentions to comfort the victim. Other than that, the results show a similar pattern: intentions to confront the bully were highest in the friendship norm condition (*M* = 4.36, *SD* = 1.33), followed by the school norm condition (*M* = 3.95, *SD* = 1.49) and control group (*M* = 3.88, *SD* = 1.40). Again, the mean difference between the friendship norm condition compared to the control group was significant (*p* = 0.035, *d* = 0.36). Mean differences between the school norm condition and control condition (*p* = 0.933), as well as the friendship norm condition and the school norm condition were non-significant (*p* = 0.078). Figure 2 displays the mean differences between the experimental conditions for the intention to comfort the victim and the intention to confront the bully.

### Effects of the Experimental Condition on the Messages to the Victim and Bully

For victim-oriented defending (i.e., sending a message to the victim in the hypothetical scenarios), the majority of the participants (54.52%) showed high levels of defending, 26.48% showed moderate levels of defending and 19.00% showed no defending. Ordinal logistic regression indicated that participants in the friendship norm condition were significantly more likely (1.8 times more likely) to show higher levels of victim-oriented defending than participants in the control condition (*B* = 0.60, *SE* = 0.27, *OR* = 1.81, *p* = 0.025). Victim-oriented defending did not differ significantly between participants in the school norm condition and participants in the control group (*B* = 0.19, *SE* = 0.26, *OR* = 1.21, *p* = 0.461). There was also no significant difference between participants in the friendship norm condition compared to the school norm condition (*B* = 0.40, *SE* = 0.27, *OR* = 1.50, *p* = 0.132; estimates based on a separate model with school norm condition as comparison group).

For bully-oriented defending (i.e., sending a message to the bully in the hypothetical scenarios), 42.68% of the participants showed high levels of defending while 28,97% showed moderate levels of defending and 28.35% showed no defending. Ordinal logistic regression revealed a similar pattern in the results as for victim-oriented defending: participants in the friendship norm condition were significantly more likely (1.71 times more likely) than participants in the control group to show higher levels of bully-oriented defending (*B* = 0.54, *SE* = 0.26, *OR* = 1.71, *p* = 0.037). There was no significant difference in bully-oriented defending in the school norm condition compared to the control group (*B* = 0.20, *SE* = 0.25, *OR* = 1.22, *p* = 0.420), nor in the friendship norm condition compared to the school norm condition (*B* = 0.34, *SE* = 0.26, *OR* = 1.40, *p* = 0.192; estimates based on a separate model with school norm condition as comparison group).

### Effects of the Experimental Condition on Participation in an Anti-Bullying Poster Campaign

Overall, participation in the anti-bullying poster campaign was low: 52.65% did not participate at all, 12.15% showed low levels of participation, 17.76% showed moderate levels of participation and 17.45% showed high levels of participation. Ordinal logistic regression revealed that there were no significant differences in participation in the anti-bullying poster campaign in the friendship norm condition compared to the control group (*B* = 0.04, *SE* = 0.26, *OR* = 1.04, *p* = 0.835), nor in the school norm condition compared to the control group (*B* = -0.32, *SE* = 0.26, *OR* = 0.73, *p* = 0.216), and the friendship norm condition compared to the school norm condition (*B* = 0.36, *SE* = 0.26, *OR* = 1.43, *p* = 0.167; estimates based on a separate model with school norm condition as comparison group).

### Robustness Analyses

#### Comprehension check

33% of participants in the experimental conditions chose the wrong answer on the valid comprehension check item. To assess whether their exclusion alters the results, the main analyses were repeated on the subset of participants who passed the comprehension check and the control group (*N* = 250). Overall, the patterns of these results are consistent with those in the full sample: the differences between the friendship-norm condition and the control condition in the intention to comfort the victim and in writing messages to both the bully and the victim remained significant in the reduced sample, and the p-values and effect sizes were highly similar to those in the full sample. The only difference was the effect on the intention to confront the bully. Here, the ANOVA was not significant in the reduced sample (*p* = 0.081; in the full sample analysis *p* = 0.027), which could be due to lower power as a consequence of the reduced sample size. Additionally, a logistic regression showed that correct (versus incorrect) responses on the comprehension check were not systematically related to participants’ gender, age, or migration background. See the Online Resource 3 for the full details on the comprehension check and all additional analyses.

#### Covariate and interaction effects

Additional models incorporating gender, age and migration background – as well as their interactions with condition – were estimated to assess the stability and generalizability of the intervention effects. See the Online Resource 4 for details. The difference between the friendship norm condition and the control group remained statistically significant for the intention to comfort the victim and for writing messages to the victim when covariates and interactions were included. For both outcomes, age emerged as a significant negative predictor. In contrast, the differences between the friendship norm condition and control group were no longer significant for the intention to confront the bully and for writing messages to the bully when the covariates and interactions were added to the models. Importantly, these changes were not driven by significant covariate or interaction effects. None of the demographic variables nor their interactions with condition reached statistical significance in these models. These findings suggest that the observed attenuation is likely due to the increased model complexity and reduced statistical power. Consistent with the primary analyses, condition had no significant effect on the intention to report the bullying incident or on the participation in the anti-bullying poster campaign.

## Discussion

(Mis-)perceptions of social norms play a powerful role in student’s behavior in bullying (Shin & Gyeong, 2024) and cyberbullying (Piccoli et al., 2020) situations. Norm influence peaks in adolescence (Laursen & Veenstra, 2021), making this a particularly interesting period to study social influence through norm perception. In the context of cyberbullying, adolescents are particularly susceptible to norms originating from groups with which they identify (Bleize et al., 2021), and friendship groups have been shown to exert strong normative influence (Bastiaensens et al., 2016). However, there is a surprising lack of norm intervention studies comparing norm influence of friendship groups with other reference groups. Thus, the current study aimed to examine the influence of friendship versus school pro-defending norms on students’ defending intentions, defending in hypothetical cyberbullying scenarios and participation in an anti-bullying poster campaign in a school-based experiment.

Results show that participants in the friendship norm condition, but not in the school norm condition, scored significantly higher than those in the control group on both defending intentions and defending in hypothetical cyberbullying scenarios, namely in form of comforting the victim and confronting the bully. The difference between the school and friendship norm condition was non-significant across measures. The experimental conditions did not affect the intention to report the cyberbullying incident or the participation in the anti-bullying poster campaign.

These results only partially reflect our initial hypotheses: Contrary to our hypotheses, participants in the school norm condition did not differ significantly from the control group on any of the outcome variables. This finding differs from the previous study by Perkins et al. (2011), in which the presentation of school norms led to a decrease in bullying and norm misperceptions. Two important differences in the intervention design may explain this discrepancy: the duration and mode of the norm presentation. Our study involved a single exposure to norm information in an online format, whereas Perkins et al. (2011) presented norm information on posters that were visible in schools for several weeks (i.e., in a shared setting). While single-session norm interventions generally show high levels of effectiveness (see Rhodes et al., 2020 for a meta-analysis), presenting norms in a shared context may be critical to increasing the impact of the intervention. The shared context ensures that participants are aware of their peers’ awareness of group norms (Paluck, 2009). For future studies, it is essential to investigate which conditions most enhance the effectiveness of norm interventions for adolescents and in the context of anti-cyberbullying interventions.

Despite these potential shortcomings of our single-exposure online norm presentation, participants in the friendship norm condition scored significantly higher than the control group on victim- and bully oriented defending intentions and defending in hypothetical cyberbullying scenarios. This finding suggests promising potential for more comprehensive interventions that address friendship norms in the context of anti-cyberbullying interventions. From a theoretical viewpoint, our results are consistent with the argument that the choice of reference group matters in norm interventions (e.g. Schultz, 2022). Traditional interventions, which are typically led by adults, become less effective during adolescence (Yeager et al., 2015). During this same period, the influence of friends increases (Laursen & Veenstra, 2021), highlighting the need to expand research on friendships as a reference group in norm interventions for adolescents. This may be especially important in the context of cyberbullying which is less visible to teachers and parents than offline settings (Peebles, 2014). Despite the clear theoretical rationale, most norm interventions to date present norms from more distal reference groups (Rhodes et al., 2020).

Beyond design-related factors, additional explanations for the lack of school norm effects may be related to adolescents’ perceptions of school as a normative group. Norms are more likely to guide behavior when individuals identify with the group that endorses them (Prentice, 2012). During adolescence, identification with broad groups such as school may be relatively weak, especially when compared to the emotional salience of friendship groups (Veenstra & Laninga-Wijnen, 2023). As a result, school norms may lack the social relevance necessary to motivate behavior change, especially in online contexts where adult supervision is limited and peer interactions often cross class, grade, and even school boundaries (Peebles, 2014). This may explain why a previous intervention in a similar context presenting class-level norms found no effects (Pfetsch et al., 2018). In contrast, friendship groups remain salient sources of influence (Bastiaensens et al., 2016), making them a particularly promising reference group for norm-based strategies targeting cyberbullying.

In addition, moral disengagement, a set of cognitive strategies that allow individuals to detach from the moral implications of harmful behavior, thereby reducing personal responsibility (Bandura, 2002), may play a critical role. Moral disengagement is typically associated with increased bullying and decreased defending behavior (Killer et al., 2019). In the context of cyberbullying, adolescents may morally disengage by employing strategies such as victim-blaming, minimizing the impact on victims, or diffusion of responsibility (Bandura, 2002). School settings may inadvertently foster moral disengagement by diffusing accountability across a large, impersonal social context. In contrast, the closeness and mutual expectations within friendship groups may reduce the likelihood of moral disengagement and promote a stronger sense of accountability to intervene.

While the friendship norm condition effectively influenced bully- and victim-oriented defending intentions and actions that reflected the specific content of the presented norms, this influence did not extend to related, but distinct behaviors such as participation in an anti-bullying poster campaign or the intention to report the cyberbullying incident. This divergence underscores the need for norm presentations to be precisely tailored to the specific behaviors they aim to change, as their effects appear limited to the behaviors directly addressed, with no spillover to related anti-bullying behaviors. Another reason for these non-significant results could be that adolescents tend to reject initiatives from adults (Yeager et al., 2018).

The observed effects ranged from small to medium, with slightly stronger effects for victim-oriented defending outcomes compared to bully-oriented ones. While modest, these effect sizes are notable given that the intervention consisted of a brief, scalable online norm presentation, requiring minimal resources. This format offers high potential for broad reach and low implementation cost. When delivered at scale, even small effects can produce meaningful real-world impact. In this study, participants were exposed to the norm information only once and in an individual online setting. Repeated exposure or presentation in a shared social context could enhance the salience and perceived relevance of the norm messages, potentially leading to stronger effects.

The study included a wide age range from late childhood to middle adolescence (10-17 years), reflecting the typical composition of a German secondary school. Age was a significant negative predictor of some outcomes (intentions to comfort the victim, sending a message to the victim, participation in the anti-bullying poster campaign), suggesting that younger participants may have been more inclined to engage in these actions regardless of condition. This is consistent with research showing that overall, defending becomes less frequent with age (Ma et al., 2019). However, none of the interactions between age and condition were statistically significant, suggesting that while there were developmental trends in defending, the effects of the friendship norm condition were not limited to a particular age subgroup. Although general susceptibility to peer influence differs across adolescence (Steinberg & Monahan, 2007), this study found that the influence of friendship norms was consistent from late childhood to middle adolescence, highlighting the impact of friendships on shaping normative behavior throughout these developmental stages.

From a practical viewpoint, there are several challenges to using friendship groups as a reference group that are reflected in our study. First, some adolescents do not have a friendship group and therefore cannot be provided with norm information. Second, there are friendship groups in which negative norms prevail, and here providing norm information could backfire and increase negative behaviors such as cyberbullying (boomerang effect, see Schultz et al., 2007). However, students in friendship groups in which pro-defending norms prevail may be the most relevant and promising addressees of such a norm intervention: defending others requires both personal and social resources (Domínguez-Hernández et al., 2018), as it is typically perceived as risky in terms of status loss and own victimization by students (Strindberg et al., 2020). Therefore, it may be students in stable and secure networks with pro-defending norms who are able to defend confidently and safely.

### Strengths and Limitations

The main strength of our study is its ecological validity due to the school setting and the use of empirically grounded norm information. The use of real attitudinal data from previous waves of data collection ensured that the norm information presented reflects the actual social realities of the participants. From an ethical perspective, the use of true norm information avoids the risk of misleading participants. However, this approach also posed unique challenges: the commitment to presenting accurate norm information led to limited participant eligibility in the friendship norm condition. Only participants who were eligible for the friendship norm condition (i.e., adolescents with friendship groups in which positive norms prevail) were included in the final sample. While this affects the generalizability of our findings, these students are the most promising targets for an intervention aimed at promoting defending.

Additionally, this data exclusion has influenced the results in a way that goes beyond generalizability, as the overall level of defending intentions and defending in the hypothetical scenarios was higher in the included sample compared to the excluded one (see Online Resource 2). The final analytic sample consisted of students embedded in supportive peer contexts where defending is more socially accepted and potentially perceived as less risky than in the general student population. Consistent with this, the final sample included a significantly higher proportion of girls (55.8%) compared to the excluded sample (42.1%), and girls are overall more likely the defend than boys (see Ma et al. 2019 for a meta-analysis). Importantly, comparisons between experimental conditions remain unaffected by this selection, as all participants in the final sample, across conditions, were eligible to the friendship norm condition, i.e., they had friendship groups in which positive norms prevail. Moreover, gender did not significantly interact with the experimental condition when predicting outcomes, showing that the experimental effects were observed in both boys and girls.

A further limitation concerns the high proportion (33%) of participants in the experimental conditions (33%) who answered the valid comprehension check item incorrectly. Importantly, follow-up analyses excluding those who failed the valid item produced results highly consistent with those observed in the full sample. Moreover, comprehension was not systematically related to participants’ age, gender, or migration background. The high failure rate likely reflects broader contextual challenges such as limited attention, fatigue, or reading difficulties. Given the educational context of the study, which included adolescents in lower-track education where reading challenges are common (Lewalter et al., 2023), excluding participants based on comprehension could undermine the ecological validity of the study (Hauser et al., 2018). Retaining all participants offers a more realistic picture of how adolescents engage with interventions in real-world school settings.

Finally, the study was conducted in a single school – an Oberschule in Lower Saxony that combines lower- and middle-track education. In Germany, early academic tracking (from grade 4) places students into distinct school types. Students in lower-track schools tend to report a more negative collective identity than their peers in higher-track schools (Knigge & Hannover, 2011), which may have reduced identification with the school and thus the impact of school norms. These contextual factors could have shaped participants’ responses to the intervention. While this school context offers valuable insight into an often underrepresented population, future studies should test the generalizability of these findings across a broader range of educational settings.

### Recommendations for Future Research

For researchers, a key takeaway from this study is that the choice of reference group in norm-based interventions is important. While social identity theory (Tajfel & Turner, 1979) suggests that norms are most influential when they originate from groups with which individuals closely identify (Schultz, 2022), most interventions to date have targeted distal reference groups (Rhodes et al., 2020). Our findings underscore the importance of selecting reference groups that are socially salient to adolescents in the context of cyberbullying. Future research should further examine how the effectiveness of norm-based interventions varies by reference group and context, particularly comparing online and offline settings.

The applied framework of this study has potential for broader applications, allowing for larger-scale investigations of the norm influence of different reference groups on defending in (cyber-)bullying situations. Given the demonstrated impact of proximal friendship norms, future research should continue to explore other peer-driven influences. For instance, popularity norms can impact how students influence each other both in terms of aggressive and prosocial behavior (Laninga-Wijnen et al., 2020), especially during adolescence when prioritization of the status goal peaks (Lafontana & Cillessen, 2010). The behavior of popular students is particularly visible and sets standards that others tend to follow in hopes of gaining social approval and greater popularity themselves (Veenstra & Lodder, 2022). Popularity norms can influence both bullying (Dijkstra & Gest, 2015) and defending (Peets et al., 2015), highlighting their potential as an important reference source in norm interventions.

Further, the role of other reference groups like teachers requires further investigation. Instead of testing their direct influence in isolation, which may be limited during adolescence (Kollerová et al., 2018), future studies could more fruitfully examine their interplay with dominant peer influences. For example, research could examine how teacher norms regarding defending are perceived, adopted, or resisted within specific friendship groups or by popular students and whether positive norms set by teachers can be amplified if they align with, or are reinforced by, influential peer groups. Additionally, studies could explore the conditions under which adult-provided norms may still gain traction among adolescents. Such work would move beyond comparisons of norm sources to understand the complex, hierarchical, and interactive nature of normative influence in adolescents’ social environments, building on the current finding that not all norm sources are equally impactful.

There are several additional ways how students can defend in cyberbullying situations online and offline. For instance, students may comment on the Instagram posts, confront the bully in person, involve a teacher or provide in-person social support for the victim. Moreover, the concrete content of the messages that are communicated towards bullies and victims can vary. It remains an important question for future research to determine which types and content of defending are most effective to reduce future cyberbullying and to improve the psychosocial adjustment of the victim.

### Practical Implications

Our findings highlight the importance of friendship norms in shaping defending in cyberbullying situations. Teachers or school counselors can address pluralistic ignorance among friends by encouraging discussions within friendship groups about defending behaviors. Such activities, aimed at reducing norm misperception among friends, would be easy to implement and would promote mutual awareness of each other’s perception of prescriptive norms. Students embedded in stable friendship groups are particularly likely to defend, making these groups effective targets for reinforcing prosocial norms. Publicly recognizing friendship groups that model defending behavior may amplify their influence as positive reference points. However, teachers need to be aware that such norm interventions can backfire (see Schultz et al., 2007) among students in friendships with negative norms. Because our findings underscore the importance of friendship groups in general, targeted intervention in these friendship groups with negative norms may also be advisable, as positive changes in these groups may have a broader impact on the school’s social environment.

In addition, designing social network interventions that account for the unique structure of friendship groups is a promising avenue. During adolescence, adult-led interventions become less effective (Yeager et al., 2015) and peer-led interventions are a promising way to develop interventions for adolescents (Veenstra, 2025). Meta-analytic evidence shows that peer-led programs can produce strong positive effects on leaders’ attitudes toward bullying (Wade et al., 2022). Embedding peer-led activities within existing friendship networks may enhance the reach and sustainability of norm change, particularly in online settings where adult monitoring is limited.

To address friendship groups in which negative norms prevail, a promising approach involves peer-led interventions, in which trained peer leaders from adjacent or overlapping social groups engage with members of negative-norm groups through collaborative projects, peer dialogue, or shared activities. Such strategies harness internal peer influence rather than relying on external adult authority, which may be resisted in adolescence (Yeager et al., 2018). Recent research suggests that negative leaders are not fundamentally different from prosocial leaders in terms of individual characteristics (Dong et al., 2023), opening up promising opportunities to shift group norms from within. Interventions that support these individuals in adopting prosocial behaviors – such as defending victims – can catalyze broader normative change across their friendship networks (Dong et al., 2023).

## Conclusion

Despite the prevalence of cyberbullying in adolescence, little is known about how the reference group of norm-based messages – whether from close friends or the broader school context – affects adolescents’ willingness to defend. To address this gap, this study compared the effects of friendship versus school pro-defending norms on adolescents’ intentions and behaviors in hypothetical cyberbullying situations. Adolescents exposed to friendship norm information were significantly more likely to endorse and enact both victim- and bully-oriented defending in hypothetical cyberbullying situations compared to those in a control group. In contrast, exposure to school-level norms did not significantly affect any outcome. These results suggest that norms from proximal, emotionally salient reference groups like friendships are more effective than norms from more distal groups like schools in motivating defending, particularly in online contexts where peer interactions are dispersed. This study demonstrates that the choice of reference group is an important design element in norm-based interventions and should be aligned with the developmental needs of adolescents. It highlights the particular importance of friendship norms in shaping defending in cyberbullying situations.

## Supplementary information

Online Resources
