# Supplementary material for: Differential Effects of Friendship and School Norms on Adolescents’ Defending in Cyberbullying Situations: A Randomized School-Based Experiment
Source: J Youth Adolesc. 2025 Jun 11;54(7):1677–92. doi: 10.1007/s10964-025-02202-y (PMC12245936; doi:10.1007/s10964-025-02202-y)
Supplement: Supplementary file 1 — Online Resources [file 10964_2025_2202_MOESM1_ESM.pdf]

Online Resources to:

Differential Effects of Friendship and School Norms on Adolescents' Defending in Cyberbullying Situations: A Randomized School-Based Experiment

Content

OR1: Pre-Intervention Analyses: Is There a Pluralistic Ignorance Effect with Respect to Defending Norms?……..…………………………………………………………….....3

Table OR1.1: Means, Standard Deviations and Results of Paired t Tests of the Perceived and Actual Prescriptive Defending Norms for Victim-Oriented and Bully-Oriented Defending at the Level of the School and Friendship Groups……………….6

OR2: Eligibility for the Friendship Norm Condition…….……….……………..……….7

Table OR2.1: Means, Standard Deviations and One-way Analyses of Variance in Defending Intentions……...………………...……………………………………….9

Table OR2.2: Ordinal Logistic Regression of Defending in Hypothetical Scenarios and Participation in an Anti-Bullying Poster Campaign…………………….………......10

Table OR2.3: Frequency Distributions for the Ordinal Measures by Eligibility to the Friendship Norm Condition (in Percentages)……………………….……..………….11

OR3: Comprehension Check……….…………………..……………………………12

Table OR3.1: Proportion of Participants who Failed the Comprehension Check per Question and per Condition………...………………...…………………………….16

Table OR3.2: Means, Standard Deviations and One-way Analyses of Variance in Defending Intentions………………..……………………………………………...17

Figure OR3.1: Mean Differences between the Experimental Conditions for the Intention to Comfort the Victim and the Intention to Confront the Bully……..……18

Table OR3.3: Ordinal Logistic Regression of Defending in Hypothetical Scenarios and Participation in an Anti-Bullying Poster Campaign…………………………………19

OR4: Covariate and Interaction Effects…………………………...…………………….....20

Table OR4.1: Fixed-Effects ANOVA Results for the Intention to Comfort the Victim……………………………………………………………………...….24

Table OR 4.2: Fixed-Effects ANOVA Results for the Intention to Confront the Bully……………………………………………………………………..….25

Table OR 4.3: Fixed-Effects ANOVA Results for the Intention to Report the Bullying Incident……………………………………………………..……………26

Table OR 4.4: Ordinal Logistic Regression Results for Writing Messages to the Victim and Bully in Hypothetical Cyberbullying Scenarios and for Participation in an Anti-bullying Poster Campaign……………………………………………….……27

References……………………………………………………..…………...30

OR1: Pre-Intervention Analyses: Is There a Pluralistic Ignorance Effect with Respect to Defending Norms?

(Mis-)perceptions of social norms play an important role in adolescents' reactions to traditional bullying (Shin & Gyeong, 2024) as well as cyberbullying situations (Bastiaensens et al., 2016). A plausible explanatory mechanism for why only few adolescents engage in defending is *pluralistic ignorance*: adolescents misinterpret their peers' inaction in bullying situations as a prescriptive norm supportive of bullying, which then contributes to their own inaction and to maintaining the status quo of low defending over time (Shin & Gyeong, 2024). In this study, information on pro-defending norms rather than (anti-)bullying norms was provided, as this may be a more direct and effective way to promote defending. The aim of these supplemental analyses is to examine whether the effect of pluralistic ignorance occurs not only with respect to bullying norms, but also with respect to defending norms, i.e., whether students misperceive their peers as less supportive of defending than they actually are. Since information on school and friendship norms was provided in the main study, it was examine whether pluralistic ignorance occurs with respect to both reference groups. For this purpose, the perceived defending norm were compared with the average defending attitude (a) at the level of the whole school and (b) at the level of the friendship group. The analyses involve the same items for victim-oriented defending and bully-oriented defending that were presented in the norm intervention in the main study.

Methods

Sample and procedure. Data stem from the second and third wave of the data collection described in the main study. In wave 2, 490 students participated (51.73% female; 22.29% with migration background (defined as participant and/or at least one parent born outside Germany); M_{age} = 12.71, SD_{age} = 1.73). In the wave 3, 498 students participated (50.70% female; 21.92% with migration background; M_{age} = 12.74, SD_{age} = 1.72). Data collection procedures were identical to those described in the main study.

Measures. The following measures were assessed at both wave 2 and wave 3.

Perceived prescriptive defending norms. Perceived prescriptive defending norms within the school (“Think about your whole school (all years and school classes). What would your schoolmates think if someone at school behaved like this?”) and the friendship group (“Think about your circle of close friends at your school. What would your close friends think if someone in your circle of friends behaved like this?”) were assessed for victim-oriented defending (“Someone comforts the victim of the bullying afterwards.“) and bully-oriented defending (“Someone tries to make the others to stop the bullying.“) on a 5-point scale ranging from 1 = “very bad” to 5 = “great”.

Defending attitudes. Defending attitudes were assessed by asking students to rate the items “One should comfort the victim of bullying afterwards.” (victim-oriented defending) and “One should try to make the others stop the bullying.” (bully-oriented defending) on a 5-point scale from 1 = “fully disagree” to 5 = “fully agree”.

For each participant, the average defending attitude within their friendship group was calculated based on their friendship nominations which were assessed with the question “Who are your friends at your school?”. Participants could nominate an unlimited number of students from a roster of names. The average victim-oriented and bully-oriented defending attitude was calculated among all nominated friends, when at least three friends (i.e., a group) were nominated.

Analyses. For each wave, paired t-tests were conducted to compare the perceived prescriptive defending norms and the average defending attitudes (a) within the whole school and (b) within the friendship groups, for both victim-oriented and bully-oriented defending.

Results

Table OR1.1 shows the means and standard deviations of the perceived prescriptive defending norms within (a) the school and (b) the friendship group, and the defending attitudes (a) across all participants within the school and (b) across the average defending

norms within participants friendship groups at wave 2 and wave 3. Perceived prescriptive defending norms were consistently lower than the corresponding actual prescriptive defending norms. In addition, perceived prescriptive school norms were consistently lower than perceived prescriptive friendship norms. Table OR1.1 also shows the results of the paired t-tests which consistently show that the perceived prescriptive defending norms are significantly lower than the corresponding actual prescriptive defending norms. Effect sizes vary between -0.35 and -0.86 and are larger for the analyses at the school level than for the analyses at the friendship group level.

Discussion

Our results show that that pluralistic ignorance with respect to defending norms occurs at both the school and friendship group level. Students perceive their peers (within their school and their friendship group) to be less supportive of defending than they actually are. Previous studies have shown that pluralistic ignorance occurs with respect to bullying norms (e.g., Perkins et al., 2011), but these analyses are the first to provide evidence of pluralistic ignorance with respect to defending norms.

Previous norm interventions targeting bullying (e.g., Tolmatcheff et al., 2022) and cyberbullying (e.g. Pfetsch et al., 2018) have typically aimed to reduce pluralistic ignorance with respect to (cyber-)bullying norms. The findings suggest that providing information about prescriptive pro-defending norms, as implemented in the main study, could be an effective strategy to promote defending.

Further, pluralistic ignorance concerning defending norms occured at both the school and friendship group level, making both reference groups a potential target group for a norm intervention to reduce pluralistic ignorance. It is important to note, however, that norms were perceived as more positive within friendship groups, so the effect of pluralistic ignorance was stronger for school norms than for friendship norms.

Table OR1.1

Means, Standard Deviations and Results of Paired t Tests of the Perceived and Actual Prescriptive Defending Norms for Victim-Oriented and Bully-Oriented Defending at the Level of the School and Friendship Groups

	Perceived norm		Actual norm					
	M	*SD*	*M*	*SD*	*t*	*df*	*p*	*d*
Wave 2								
Victim-oriented defending								
School	3.84	0.93	4.68	0.59	-18.98	486	<.001	-0.86
Friendship groups	4.24	0.90	4.70	0.32	-8.70	341	<.001	-0.47
Bully-oriented defending								
School	3.85	1.03	4.62	0.70	-15.14	486	<.001	-0.69
Friendship groups	4.22	0.92	4.65	0.33	-7.41	341	<.001	-0.40
Wave 3								
Victim-oriented defending								
School	3.84	0.93	4.66	0.68	-19.13	495	<.001	-0.86
Friendship groups	4.24	0.89	4.70	0.33	-7.45	345	<.001	-0.40
Bully-oriented defending								
School	3.81	0.99	4.55	0.77	-16.20	495	<.001	-0.73
Friendship groups	4.17	0.94	4.60	0.35	-6.48	345	<.001	-0.35

Note. Data on the actual defending norms within the friendship group was available for 342 participants at wave 2 and 346 participants at wave 3.

OR2: Eligibility for the Friendship Norm Condition

The study involved a school experiment carried out in a naturalistic setting in which only true information about prescriptive norms was presented. The experiment was implemented in the framework of the fourth wave of a longitudinal school-wide survey. Norm information was based on participants responses from the second and third waves of data collection at the school. As a consequence of this experimental design, participants were only eligible for the friendship norm condition if they met all of the following conditions:

(1) they participate in at least one wave of the previous data collection, and therefore information about their friendship group was available

(2) they reported at least three friends in either wave of the previous data collection and thus had a sufficiently large friendship group

(3) their friendship group met the criteria for reporting positive prescriptive defending norms (above 93% agreement with pro-defending attitudes).

Our initial sample consisted of 496 participants who were randomly and with equal probability assigned to one of the three experimental conditions. Two participants did not respond to any of the outcome variables and was therefore excluded from the analyses. Participants who were assigned to, but not eligible for, the friendship norm condition were re-assigned randomly and with equal probability to one of the two remaining conditions. This resulted in the following distribution between the conditions: 196 participants in the control condition, 193 participants in the school norm condition, and 105 participants in the friendship norm condition.

To avoid systematic differences between the friendship norm condition and the other conditions due to the limited eligibility for the friendship norm condition, preliminary analyses were conducted to check whether eligibility for the friendship norm condition had a significant main effect on any of the dependent variables.

In the preregistration of the preliminary analyses, it was proposed to examine whether there was a significant main effect of the eligibility for the friendship norm condition or an interaction effect between the eligibility for the friendship norm condition and the experimental condition on any of the outcome variables. However, eligibility for the friendship norm condition and the experimental condition are not independent of each other, and adding both variables as predictors in the same model would have led to estimation problems. Therefore, the main effect of the eligibility for the friendship norm condition on the outcome variables was tested.

To test the effect of the eligibility for the friendship norm condition on defending intentions (comforting the victim; confronting the bully; reporting the bullying incident), a MANOVA was conducted. The analysis revealed an overall significant effect of the eligibility for the friendship norm condition on the combined dependent variables of defending intentions ($F(3,490) = 5.45$, $p = 0.001$. Follow-up univariate ANOVAs were performed. The results are shown in Table OR2.1.

To test the effect of the eligibility for the friendship norm condition on defending by sending a message to the victim and the bully in hypothetical scenarios, as well as on willingness to participate in an anti-bullying poster campaign, three ordinal logistic regressions were run See Table OR2.2 for the results and Table OR2.3 for the frequency distributions of the ordinal measures by eligibility for the friendship norm condition.

A significant main effect of eligibility for the friendship norm condition was found across all outcome variables. In accordance with the preregistered guidelines, participants who were not eligible for the friendship norm condition were excluded from the main analyses to avoid systematic variation between the three conditions. Consequently, the final sample consisted of 321 participants with the following distribution between the conditions: 106 participants in the control condition, 110 participants in the school norm condition, and 105 participants in the friendship norm condition.

Table OR2.1

Means, Standard Deviations and One-way Analyses of Variance in Defending Intentions

Variable	Eligible to friendship norm condition (N = 321)		Non-eligible to friendship norm condition (N = 174)		*F(1,492)*
	M	*SD*	*M*	*SD*	
Comfort the victim	4.52	1.38	4.00	1.50	14.56***
Confront the bully	4.06	1.42	3.62	1.56	9.96**
Report the bullying	4.07	1.66	3.69	1.74	5.76*

Note. * p < .05; ** p < .01; *** p < .001.

Table OR2.2

Ordinal Logistic Regression of Defending in Hypothetical Scenarios and Participation in an Anti-Bullying Poster Campaign

Variable / Comparison	*Estimate*	*SE*	*t*
Message to the victim			
Eligible vs. non-eligible to friendship norm condition	0.64	0.18	3.60***
Message to the bully			
Eligible vs. non-eligible to friendship norm condition	0.48	0.18	2.73**
Participation poster-campaign			
Eligible vs. non-eligible to friendship norm condition	0.57	0.19	3.02**

Note. ** p < .01; *** p < .001.

Table OR2.3

Frequency Distributions for the Ordinal Measures by Eligibility to the Friendship Norm Condition (in Percentages)

	Defending in hypothetical scenarios			
	None	Moderate level	High level	
Message to the victim				
Eligible	19.0%	26.5%	54.5%	
Non-eligible	33.5%	26.0%	40.5%	
Message to the bully				
Eligible	28.3%	29.0%	42.7%	
Non-eligible	40.5%	26.6%	32.9%	
	Participation			
	None	Low level	Moderate level	High level
Poster campaign				
Eligible	52.6%	12.1%	17.8%	17.4%
Non-eligible	67.1%	8.1%	13.9%	11.0%

Note. The percentages represent the proportion of eligible vs non-eligible students engaging in bully- and victim-oriented defending in hypothetical scenarios and the poster campaign.

OR3: Comprehension Check

A factual manipulation check (Kane & Barabas, 2019) with two single-choice questions (each one representing one comprehension check) was assessed at the end of the questionnaire. First, participants were asked “Whose average answers did we present to you?” with response categories 1 = “results from your friends” and 2 = “results of the students in your school”. Second, they were asked “How large was the agreement with the statements that were presented to you?” with response categories 1 = “less than 10% (few people agreed)”, 2 = “about 50% (about half agreed)” and 3 = “more than 90% (almost everyone agreed)”.

The proportion of students who failed the comprehension check (i.e., they answered incorrectly to at least one of the two questions) was very high: 215 out of the 321 participants were asked to complete the comprehension check (while the remaining 106 students were in the control condition). Of these 215 participants, 109 failed the comprehension check (51%). For the first question, the proportion of incorrect answers was 32%. For the second question, the proportion of incorrect answers was 33%. Table OR3.1 shows the proportion of participants who failed the comprehension check per question and per condition.

The high proportion of incorrect answers indicates potential problems in the formulation and implementation of the comprehension check task. A mistake was identified in the wording of the first question: participants were asked “Whose average answers did we present to you?” with the response options “results from your friends” and “results of the students in your school”. Participants assigned to school norms were supposed to choose “the students in your school” and participants assigned to friendship norms were supposed to choose “friends”. However, the friends referred to in this study are always students in their school. As the two response options do not allow for a clear distinction between conditions, this question cannot be used. The second question check appears unambiguous: here, participants were asked “How large was the agreement with the statements that were

presented to you?” with the response options “less than 10% (few people agreed)”, “about 50% (about half agreed)” and “more than 90% (almost everyone agreed)”.

Accordingly, although initially two comprehension checks were included, only one is valid to be used. On this remaining question, 33% of participants choose the wrong answer for their condition. To assess whether their exclusion would alter the findings, additional analyses were conducted on the subset of participants who passed the second comprehension check and the control group ($N = 250$). This approach is in line with recommendations by Hauser et al. (2018). See Table OR3.2 and Figure OR3.1 for the results of the effect of the experimental condition on the measures of behavioural intentions and Table OR3.3 for the results of the effect of the experimental condition on the measures of defending in a hypothetical scenario and participation in an anti-bullying poster campaign in the reduced sample of students who did not fail the second item of the comprehension check.

Overall, the patterns of these results are consistent with those in the full sample: the differences between the friendship-norm condition and the control condition in the intention to comfort the victim and in writing messages to both the bully and the victim remained significant in the reduced sample, and the p-values of the post-hoc tests and ordinal logistic regressions were highly similar to those in the full sample (intention to comfort the victim: $p < .001$ in the full sample, $p = 0.004$ in the reduced sample; victim-oriented defending in hypothetical scenarios: $p = 0.025$ in the full sample, $p = 0.012$ in the reduced sample; bully- oriented defending in hypothetical scenarios: $p = 0.037$ in the full sample, $p = 0.043$ in the reduced sample). The only difference was the effect on the intention to confront the bully. Here, the ANOVA was not significant in the reduced sample ($p = 0.081$; in the full sample analysis $p = 0.027$), which could be due to lower power as a consequence of the reduced sample size.

The effect sizes also were highly similar (intention to comfort the victim: $d = 0.51$ in the full and reduced sample; intention to confront the bully: $d = 0.36$ in the full sample, $d =$

0.35 in the reduced sample; victim-oriented defending in hypothetical scenarios: *OR* = 1.81 in the full sample, *OR* = 2.20 in the reduced sample; bully- oriented defending in hypothetical scenarios: *OR* = 1.71 in the full sample *OR* = 1.82 in the reduced sample).

To examine whether demographic characteristics were associated with performance on the second comprehension check item, a logistic regression analysis was conducted with the correctness of response (correct vs. incorrect) as the binary outcome variable. The predictors included gender, age (centered), and migration background. Participants who indicated "diverse" for gender ($N = 2$) were coded as missing. The overall model did not significantly improve fit compared to the null model ($\chi 2(3) = 0.96$, $p = .812$), and none of the predictors were statistically significant: Gender was not associated with the likelihood of giving a correct response ($\beta = 0.01$, $SE = 0.30$, $z = 0.03$, $p = .978$). Age also showed no significant association with correct versus incorrect responses ($\beta = 0.07$, $SE = 0.09$, $z = 0.83$, $p = .406$). Similarly, migration background was unrelated to correctness of response ($\beta = -0.178$, $SE = 0.374$, $z = -0.477$, $p = .634$). These results show that correct (versus incorrect) responses on the comprehension check were not systematically related to participants' gender, age, or migration background.

Additionally, a logistic regression was conducted to test whether sex, age or migration background predicted a wrong answer compared to a correct answer to the second comprehension check. None of these predictors were statistically significant, suggesting that a wrong answer to the comprehension check is not systematically related to these demographical characteristics.

The high error rate suggests that there were problems related to the comprehension check that go beyond a lack of understanding of the study materials, such as a general misunderstanding of the comprehension check task, lack of attention or fatigue. This is supported by the observations reported by the research assistants who were present during the

data collection: many students were confused by the comprehension check and asked very general questions such as “What am I supposed to do here?”.

The aim of the present study was to examine the effects of a norm intervention on adolescents’ reactions to hypothetical cyberbullying scenarios in a school setting, which inevitably involves varying levels of attention, motivation, and comprehension - especially within a sample of adolescents attending lower-track education, many of whom have reading difficulties (Lewalter et al., 2023). Adolescents in lower track education are generally an understudied population, and intervention studies with this population are severely lacking. Excluding participants based on the comprehension check would risk discarding real-world variability in how adolescents engage with such interventions. Removing these participants could compromise the ecological validity of the findings (Hauser et al., 2018). Most importantly, the highly similar results between the full sample and the reduced samples underscore that the presence or absence of correct comprehension check responses does not fundamentally alter the conclusions. Consequently, all participants were retained in the primary analyses presented in the main article.

Table OR3.1

Proportion of Participants who Failed the Comprehension Check per Question and per Condition

condition	Question 1		Question 2	
	Correct	incorrect	Correct	incorrect
School norm	79	31	77	33
Friendship norm	68	37	67	38
Control	-	-	-	-

Note. Frequencies are displayed in absolute numbers.

Table OR3.2

Means, Standard Deviations and One-way Analyses of Variance in Defending Intentions

Variable	school		friendship		control		*F(2,247)*
	M	*SD*	*M*	*SD*	*M*	*SD*	
Comfort the victim	4.59	1.41	4.90	1.20	4.20	1.43	5.49**
Confront the bully	4.13	1.46	4.37	1.42	3.88	1.40	2.54
Report the bullying	4.21	1.67	4.57	1.53	4.01	1.64	2.46

Note. ** $p < .01$. $N = 250$ (participants who failed the comprehension check item “How large was the agreement with the statements that were presented to you?” were excluded from the analysis).

a. Defending intention to comfort the victim

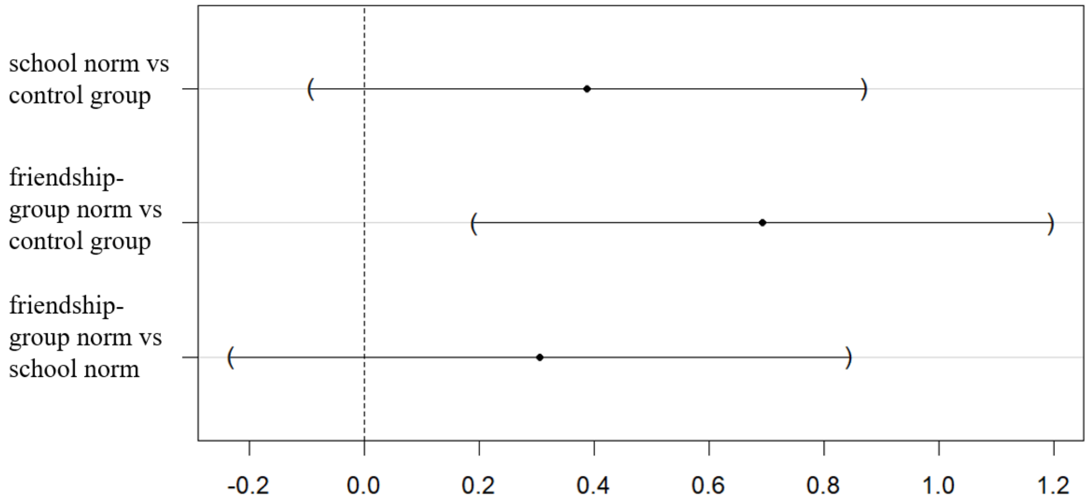

b. Defending intention to confront the bully

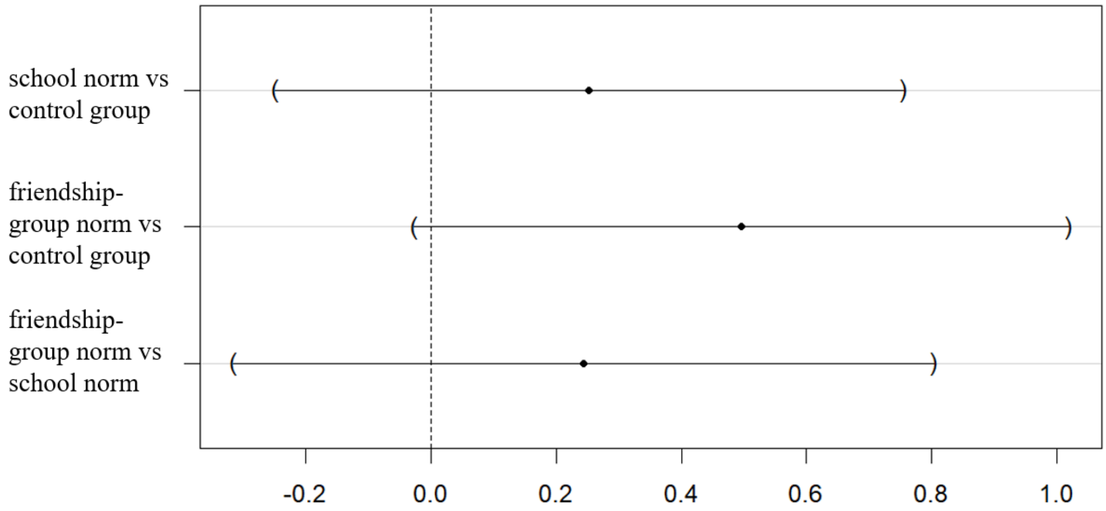

Fig OR3.1 Mean differences between pairs of experimental conditions with 95% confidence intervals for (a) the intention to comfort the victim and (b) the intention to confront the bully. $N = 250$ (participants who failed the comprehension check item “How large was the agreement with the statements that were presented to you?” were excluded from the analysis)

Table OR3.3

Ordinal Logistic Regression of Defending in Hypothetical Scenarios and Participation in an Anti-Bullying Poster Campaign

Variable / Comparison	*Estimate*	*SE*	*t*
Message to the victim			
school vs control	0.34	0.29	1.19
friendship group vs control	0.79	0.31	2.51*
friendship group vs school [a]	0.44	0.34	1.30
Message to the bully			
school vs control	0.47	0.28	1.68
friendship group vs control	0.60	0.30	2.02*
friendship group vs school [a]	0.14	0.32	0.43
Participation poster-campaign			
school vs control	-0.22	0.28	-0.79
friendship group vs control	-0.15	0.30	-0.49
friendship group vs school [a]	0.07	0.32	0.23

Note. [a] estimates based on a separate model with school norm condition as comparison group. * $p < .05$. $N = 250$ (participants who failed the comprehension check item “How large was the agreement with the statements that were presented to you?” were excluded from the analysis).

OR4: Covariate and interaction effects

To examine the robustness of the main findings, a series of additional analyses were conducted that included age, gender, and migration background, as well as their interactions with condition, as predictors in all models. Age was centered prior to analysis to facilitate the interpretation of interaction terms.

Effects of the Experimental Condition on Defending Intentions

A MANOVA was conducted to examine the effects of condition, gender, age (centered), and migration background, as well as their interactions with condition, on the combined dependent variables intention to comfort the victim, intention to confront the bully and intention to report the bullying incident. The analysis revealed statistically significant multivariate effects of condition, ($F(6, 612) = 2.63$, $p = .016$) and age ($F(3, 305) = 11.44$, $p < .001$). No significant multivariate effects were found for gender ($F(3, 305) = 1.27$, $p = .284$), migration background ($F(3, 305) = 0.99$, $p = .397$), and the interaction terms condition × gender ($F(6, 612) = 0.71$, $p = .645$), condition × age ($F(6, 612) = 0.98$, $p = .436$) and condition × migration background ($F(6, 612) = 0.42$, $p = .864$).

The results of follow-up univariate ANOVAs are displayed in Table OR4.1 for the intention to comfort this victim, Table OR4.2 for the intention to confront the bully and OR4.3 for the intention to report the bullying incident. Consistent with the analyses reported in the main article, condition had a significant effect on the intention to comfort the victim. In addition, age was a significant predictor of the intention to comfort the victim, while gender, migration background, and all interaction effects were non-significant. Regarding the intention to confront the bully, none of the predictors reached statistical significance. Notably, the significant effect of condition reported in the main article did not remain significant when additional covariates and interaction terms were included in the model ($p = .084$). Similarly, for the intention to report the bullying incident, no significant effects were found for any of the predictors, which is in line with the findings reported in the main paper.

Post-hoc comparisons using Tukey-adjusted estimated marginal means were conducted to further examine the effect of condition on the intention to comfort the victim. These comparisons are based on estimated marginal means, adjusted for age (centered at its mean) and averaged over levels of sex and migration background. Although the model included interaction terms between condition and covariates, none were statistically significant, and the results presented here reflect the main effect of condition on comfort intention. Participants in the friendship norm condition ($M = 4.76$, $SE = 0.18$) reported significantly higher intentions to comfort the victim than those in the control condition ($M = 4.20$, $SE = 0.15$), $p = .044$. No significant differences were found between the control and school norm conditions ($M = 4.41$, $SE = 0.17$; $p = .614$) or between the school norm and friendship norm condition ($p = .330$). These results are in line with the pattern reported in the main article.

For the intention to confront the bully were calculated for each condition, estimated marginal means were again computed controlling for age (centered at its mean) and averaged over levels of sex and migration background. Participants in the control condition reported the lowest intention to confront ($M = 3.88$, $SE = 0.16$), followed by the school norm condition ($M = 4.08$, $SE = 0.18$), and the friendship norm condition ($M = 4.29$, $SE = 0.19$). Tukey-adjusted post-hoc comparisons indicated that the differences between conditions were not statistically significant: control vs. school norm ($p = .674$), control vs. friendship norm ($p = .207$), and school norm vs. friendship norm ($p = .681$). This contrasts with the findings reported in the main article, where the comparison between the control and friendship norm conditions was statistically significant when analyzed without covariates and interactions. Although none of the additional predictors or interaction terms were statistically significant, their inclusion still influenced the model. First, adding covariates increases model complexity, which can raise residual variance and reduce statistical power, making it harder to detect effects that appeared significant in simpler models. Second, some covariates may share variance with condition,

even without reaching significance themselves. This partial overlap can reduce the unique variance attributed to condition, resulting in more conservative estimates. Together, these factors likely contributed to the non-significant condition effect observed in the extended model.

Effects of the Experimental Condition on the Messages to the Bully and Victim and the Participation in an Anti-Bullying Poster Campaign

Ordinal logistic regressions were conducted to examine participants' responses in hypothetical cyberbullying scenarios (i.e., writing messages to the bully and victim). Each model included condition, gender, age (centered) and migration background as predictors, along with interaction terms between condition and each covariate. As condition comprised three levels, all models were estimated twice: first with the control condition as the reference category, and then with the school norm condition as the reference category, to allow for direct comparisons between the school norm and friendship norm condition. The results of these analyses are presented in Table OR4.4.

Participants in the friendship norm condition were significantly more likely to send messages to the victim compared to those in the control condition (OR = 2.53). Comparisons between the school norm condition and control condition, as well as the friendship norm condition and school norm condition were not statistically significant. This is in line with the results reported in the main article. Age was also a significant predictor in this model, with older participants being less likely to write a message to the victim. No other main effects or interaction terms reached statistical significance.

In the model predicting whether participants wrote messages to the bully, no statistically significant effects were found for condition or any of the covariates or interaction terms. Participants in the friendship norm condition were descriptively more likely send messages to the bully compared to those in the control condition (OR = 1.91, p = 0.090), and also compared to those in the school norm condition (OR = 1.95 p = .073), but these

differences were not statistically significant. As for the intention to confront the bully, this contrasts with the findings reported in the main article, where the comparison between the control and friendship norm conditions was statistically significant when analyzed without covariates and interactions. These results diverge from those reported in the main article, where the comparison between the friendship norm and control conditions yielded a significant effect in predicting writing messages to the bully. Again, this discrepancy likely reflects the inclusion of additional covariates and interaction terms in the current models. These extended models provide a more conservative test of the condition effect by accounting for individual differences that may share variance with the experimental manipulation. Although these covariates and interactions did not reach significance themselves, their inclusion increases model complexity and residual variance, which can reduce statistical power. This may have attenuated the previously observed effect of condition on writing messages to the bully.

In the models predicting participation in the anti-bullying poster campaign, no significant effects were observed for condition. This is consistent with the results reported in the main article. Age again emerged as a significant negative predictor, indicating that older students were less likely to participate. No other main effects or interaction terms reached statistical significance.

Conclusion

Taken together, the results of these additional analyses indicate that the key findings reported in the main article remain robust when controlling for age, gender, and migration background. While the effects related to confronting the bully were attenuated in the extended models, the overall pattern remained consistent. Importantly, none of the interaction terms between condition and the demographic covariates reached statistical significance across all models. This suggests that the effects of the intervention were not moderated by age, gender, or migration background, indicating a similar pattern of responsiveness across subgroups.

Table OR4.1

Fixed-Effects ANOVA Results for the Intention to Comfort the Victim

Predictor	Sum of Squares	*df*	Mean Square	*F*	*p*	partial η^2
intercept	786.47	1	786.47	446.65	.000	
condition	17.76	2	8.88	5.04	.007	.03
gender	0.02	1	0.02	0.01	.921	.00
age	12.71	1	12.71	7.22	.008	.02
migration background	0.08	1	0.08	0.05	.828	.00
condition x gender	3.42	2	1.71	0.97	.380	.01
condition x age	2.43	2	1.22	0.69	.502	.00
condition x migration background	0.05	2	0.02	0.01	.987	.00
error	540.57	307	1.76			

Note. Age was mean-centered.

Table OR4.2

Fixed-Effects ANOVA Results for the Intention to Confront the Bully

Predictor	Sum of Squares	*df*	Mean Square	*F*	*p*	partial η^2
intercept	719.94	1	719.94	364.76	.000	
condition	9.84	2	4.92	2.49	.084	.02
gender	2.52	1	2.52	1.27	.260	.00
age	4.07	1	4.07	2.06	.152	.01
migration background	0.00	1	0.00	0.00	.983	.00
condition x gender	1.31	2	0.66	0.33	.717	.00
condition x age	2.62	2	1.31	0.66	.515	.00
condition x migration background	2.10	2	1.05	0.53	.588	.00
error	605.94	307	1.97			

Note. Age was mean-centered.

Table OR4.3

Fixed-Effects ANOVA Results for the Intention to Report the Bullying Incident

Predictor	Sum of Squares	*df*	Mean Square	*F*	*p*	partial η^2
intercept	816.23	1	816.23	300.34	.000	
condition	12.38	2	6.19	2.28	.104	.01
gender	4.89	1	4.89	1.80	.181	.01
age	6.59	1	6.59	2.43	.120	.01
migration background	2.06	1	2.06	0.76	.385	.00
condition x gender	3.82	2	1.91	0.70	.496	.00
condition x age	1.38	2	0.69	0.25	.776	.00
condition x migration background	3.08	2	1.54	0.57	.568	.00
error	834.34	307	2.72			

Note. Age was mean-centered.

Table OR4.4

Ordinal Logistic Regression Results for Writing Messages to the Victim and Bully in Hypothetical Cyberbullying Scenarios and for Participation in an Anti-bullying Poster Campaign

Variable / Comparison	*Estimate*	*SE*	*t*
Message to the victim			
Reference category: control			
school norm vs control	0.36	0.42	0.86*
friendship norm vs control	0.93	0.42	2.23
gender	-0.27	0.37	-0.74
age	-0.24	0.10	-2.29*
migration background	-0.38	0.41	-0.92
school norm * gender	-0.37	0.54	-0.69
friendship norm * gender	-0.77	0.55	-1.39
school norm * age	-0.15	0.15	-0.99
friendship norm * age	0.11	0.16	0.72
school norm * migration background	0.07	0.64	0.10
friendship norm * migration background	-0.37	0.65	-0.58
Reference category: school norm			
control vs school norm	-0.36	0.42	-0.86
friendship norm vs school norm	0.57	0.42	1.34
gender	-0.64	0.39	-1.66
age	-0.39	0.12	-3.37***
migration background	-0.31	0.49	-0.64
control * gender	0.37	0.54	0.69
friendship norm * gender	-0.40	0.56	-0.70
control * age	0.15	0.15	0.99
friendship norm * age	0.26	0.16	1.62
control * migration background	-0.07	0.64	-0.10
friendship norm * migration background	-0.44	0.70	-0.63
Message to the bully			
Reference category: control			
school norm vs control	-0.02	0.38	-0.06
friendship norm vs control	0.65	0.38	1.70
gender	-0.42	0.36	-1.18

age	-0.09	0.10	-0.92
migration background	-0.14	0.41	-0.33
school norm * gender	0.22	0.50	0.43
friendship norm * gender	-0.50	0.53	-0.94
school norm * age	0.07	0.15	0.45
friendship norm * age	-0.03	0.15	-0.21
school norm * migration background	0.52	0.62	0.84
friendship norm * migration background	0.10	0.63	0.16
Reference category: school norm			
control vs school norm	0.02	0.38	0.06
friendship norm vs school norm	0.67	0.37	1.80
gender	-0.20	0.36	-0.56
age	-0.02	0.11	-0.25
migration background	0.39	0.47	0.82
control * gender	-0.22	0.50	-0.43
friendship norm * gender	-0.72	0.53	-1.35
control * age	-0.07	0.15	-0.45
friendship norm * age	-0.10	0.15	-0.64
control * migration background	-0.52	0.62	-0.84
friendship norm * migration background	-0.42	0.67	-0.62
Participation poster-campaign			
Reference category: control			
school norm vs control	-0.43	0.41	-1.05
friendship norm vs control	-0.02	0.39	-0.04
gender	-0.27	0.38	-0.71
age	-0.23	0.11	-2.14*
migration background	0.43	0.44	0.97
school norm * gender	0.40	0.53	0.76
friendship norm * gender	0.09	0.55	0.16
school norm * age	0.05	0.15	0.30
friendship norm * age	0.01	0.16	0.10
school norm * migration background	-0.29	0.66	-0.43
friendship norm * migration background	0.21	0.64	0.33
Reference category: school norm			
control vs school norm	0.43	0.41	1.05

	friendship norm vs school norm	0.41	0.37	1.10
	gender	0.13	0.37	0.36
	age	-0.18	0.11	-1.64
	migration background	0.14	0.50	0.28
	control * gender	-0.40	0.53	-0.76
	friendship norm * gender	-0.31	0.54	-0.58
	control * age	-0.05	0.15	-0.30
	friendship norm * age	-0.03	0.16	-0.120
	control * migration background	0.29	0.66	0.43
	friendship norm * migration background	0.49	0.68	0.72

Note. Age was mean-centered. * $p < .05$; *** $p < .001$.

References

Bastiaensens, S., Pabian, S., Vandebosch, H., Poels, K., Van Cleemput, K., Desmet, A., & De Bourdeaudhuij, I. (2016). From normative influence to social pressure: How relevant others affect whether bystanders join in cyberbullying. *Social Development*, *25*(1), 193–211. https://doi.org/10.1111/sode.12134

Hauser, D. J., Ellsworth, P. C., & Gonzalez, R. (2018). Are manipulation checks necessary? *Frontiers in Psychology*, *9*. https://doi.org/10.3389/fpsyg.2018.00998

Kane, J. V., & Barabas, J. (2019). No harm in checking: Using factual manipulation checks to assess attentiveness in experiments. *American Journal of Political Science*, *63*(1), 234–249. https://doi.org/10.1111/ajps.12396

Lewalter, D., Diedrich, J., Goldhammer, F., Köller, O., & Reiss, K. (Eds.). (2023). *PISA 2022*. Waxmann Verlag GmbH. https://doi.org/10.31244/9783830998488

Perkins, H. W., Craig, D. W., & Perkins, J. M. (2011). Using social norms to reduce bullying: A research intervention among adolescents in five middle schools. *Group Processes and Intergroup Relations*, *14*(5), 703–722. https://doi.org/10.1177/1368430210398004

Pfetsch, J., Schultze-Krumbholz, A., & Füllgraf, F. (2018). Does the information about classroom norms change the individual injunctive norms about cyberbullying? A minimal intervention study. *International Journal of Developmental Sciences*, *12*(3–4), 147–157. https://doi.org/10.3233/DEV-180250

Shin, H., & Gyeong, S. (2024). Perceived and actual norms, and norm misperceptions in explaining participant roles in bullying. *Journal of Youth and Adolescence*. https://doi.org/10.1007/s10964-024-02042-2

Tolmatcheff, C., Galand, B., Roskam, I., & Veenstra, R. (2022). The effectiveness of moral disengagement and social norms as anti-bullying components: A randomized controlled trial. *Child Development*, *93*(6), 1873–1888. https://doi.org/10.1111/cdev.13828
